# Inorganic carbon and nitrogen assimilation in cellular compartments of a benthic kleptoplastic foraminifer

**DOI:** 10.1038/s41598-018-28455-1

**Published:** 2018-07-04

**Authors:** Charlotte LeKieffre, Thierry Jauffrais, Emmanuelle Geslin, Bruno Jesus, Joan M. Bernhard, Maria-Evangelia Giovani, Anders Meibom

**Affiliations:** 1 Laboratory for Biological Geochemistry, School of Architecture, Civil and Environmental Engineering (ENAC), Ecole Polytechnique Fédérale de Lausanne (EPFL), 1015 Lausanne, Switzerland; 2 UMR CNRS 6112 LPG-BIAF, Université d’Angers, 2 Boulevard Lavoisier, 49045 Angers, CEDEX 1 France; 3 EA2160, Laboratoire Mer Molécules Santé, Université de Nantes, Nantes, France; 4 BioISI – Biosystems & Integrative Sciences Institute, Campo Grande University of Lisboa, Faculty of Sciences, Lisboa, Portugal; 5 Woods Hole Oceanographic Institution, Department of Geology & Geophysics, Woods Hole, MA USA; 6 Center for Advanced Surface Analysis, Institute of Earth Sciences, University of Lausanne, 1015 Lausanne, Switzerland

## Abstract

*Haynesina germanica*, an ubiquitous benthic foraminifer in intertidal mudflats, has the remarkable ability to isolate, sequester, and use chloroplasts from microalgae. The photosynthetic functionality of these kleptoplasts has been demonstrated by measuring photosystem II quantum efficiency and O_2_ production rates, but the precise role of the kleptoplasts in foraminiferal metabolism is poorly understood. Thus, the mechanism and dynamics of C and N assimilation and translocation from the kleptoplasts to the foraminiferal host requires study. The objective of this study was to investigate, using correlated TEM and NanoSIMS imaging, the assimilation of inorganic C and N (here ammonium, NH_4_^+^) in individuals of a kleptoplastic benthic foraminiferal species. *H*. *germanica* specimens were incubated for 20 h in artificial seawater enriched with H^13^CO_3_^−^ and ^15^NH_4_^+^ during a light/dark cycle. All specimens (n = 12) incorporated ^13^C into their endoplasm stored primarily in the form of lipid droplets. A control incubation in darkness resulted in no ^13^C-uptake, strongly suggesting that photosynthesis is the process dominating inorganic C assimilation. Ammonium assimilation was observed both with and without light, with diffuse ^15^N-enrichment throughout the cytoplasm and distinct ^15^N-hotspots in fibrillar vesicles, electron-opaque bodies, tubulin paracrystals, bacterial associates, and, rarely and at moderate levels, in kleptoplasts. The latter observation might indicate that the kleptoplasts are involved in N assimilation. However, the higher N assimilation observed in the foraminiferal endoplasm incubated without light suggests that another cytoplasmic pathway is dominant, at least in darkness. This study clearly shows the advantage provided by the kleptoplasts as an additional source of carbon and provides observations of ammonium uptake by the foraminiferal cell.

## Introduction

Kleptoplasty is defined as the process in which a cell sequesters algal chloroplasts while discarding or digesting other algal components^1^. This phenomenon is encountered in different organisms, such as sacoglossans (sea slugs)^2–4^, ciliates^5^, dinoflagellates^6–8^, and benthic foraminifera^9,10^.

Studies of benthic foraminiferal kleptoplasty have focused on shallow-water species inhabiting photic zones, especially *Haynesina germanica* and *Elphidium* spp. These studies have relied on ultrastructural observations and/or genetic analyses, which established the diatom origin of the kleptoplasts, or incubation/starvation experiments to define kleptoplast lifetimes and functionality once inside the foraminiferal cells^10–21^. Additionally^22^, showed that *H*. *germanica* and *Elphidium williamsoni* had a net uptake of inorganic carbon (H^14^CO_3_) in light, and experiments with oxygen microelectrodes demonstrated that maximal O_2_ production by *H*. *germanica* depended on light intensity and light history^11,23^. A kleptoplastic strategy thus provides these organisms with both carbon and a source of oxygen. Cesbron *et al*.^11^ hypothesized that kleptoplasts might constitute an additional carbon source that may provide the kleptoplastic foraminifera a substantial competitive advantage, especially during periods of impoverished nutrients. However, the extent to which kleptoplasty contributes to the carbon within the foraminiferal cell storage via photosynthetic C assimilation has not been studied yet.

Foraminiferal kleptoplasts might also be involved in uptake of inorganic N. Indeed, diatoms, from which foraminifera sequester their kleptoplasts^20,24,25^, are able to assimilate ammonium through the chloroplast GS/GOGAT (glutamate synthase and glutamine oxoglutarate aminotransferase) enzymatic pathway^26–28^. Kleptoplasty is also shown in deep-sea species^9,24,25,29^ living in complete darkness and thus unable to perform photosynthesis^9,24^. Among these deep-sea species, *Nonionella stella* maintains kleptoplasts and associated functional enzymatic machinery, including ribulose bis-phosphate carboxylase oxygenase (RuBisCO) and phosphoenol pyruvate carboxylase (PEP carboxylase), intact for months in the dark after sampling^24^. It was suggested that kleptoplasts in these deep-sea species are involved in assimilation of inorganic N^24^. Therefore, a similar role in shallow kleptoplastic foraminiferal species is possible and needs to be investigated.

To date, no studies have documented the timing and distribution of assimilation and translocation of C-photosynthates or N-compounds between kleptoplasts and the foraminiferal cell. To precisely trace the C and N assimilation within the different cell compartments in the kleptoplastic foraminiferal cell, the NanoSIMS (nanoscale secondary ion mass spectrometry, a relatively recent ultra-high resolution isotopic imaging method^30,31^), was used in combination with transmission electron microscopy (TEM) and stable isotope labeling experiments^30,31^. This combined approach has already been successfully applied to study assimilation, storage, and transfer of C and N in several different marine organisms^32–39^, including foraminifera^40–43^. Using this integrative approach, the present study had three objectives: (1) investigate the role of kleptoplasts in C-fixation, (2) investigate the transfer and distribution of photosynthetically produced organic C within the host; and (3) investigate the potential role of kleptoplasts in foraminiferal N metabolism.

## Results

**Figure 1:**
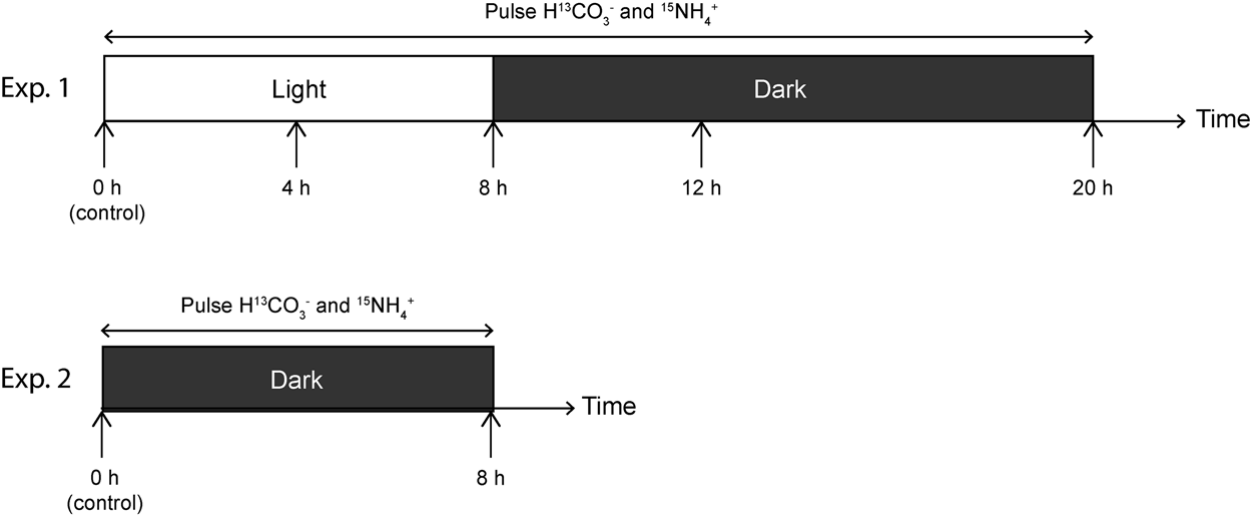
Schematic of Experiments 1 and 2, exposing *H*. *germanica* to different light conditions. Three specimens were sampled at each indicated time point. See text for details.

*H*. *germanica* specimens were incubated for 20 h in artificial seawater enriched with 2 mM NaH^13^CO_3_ and 10 μM ^15^NH_4_Cl, following a light - dark cycle (Fig. 1, see details in Methods). Specimens were preserved for analysis at regular time intervals (i.e., after 4, 8, 12, and 20 hours).

### TEM observations of foraminiferal cytoplasm

**Figure 2:**
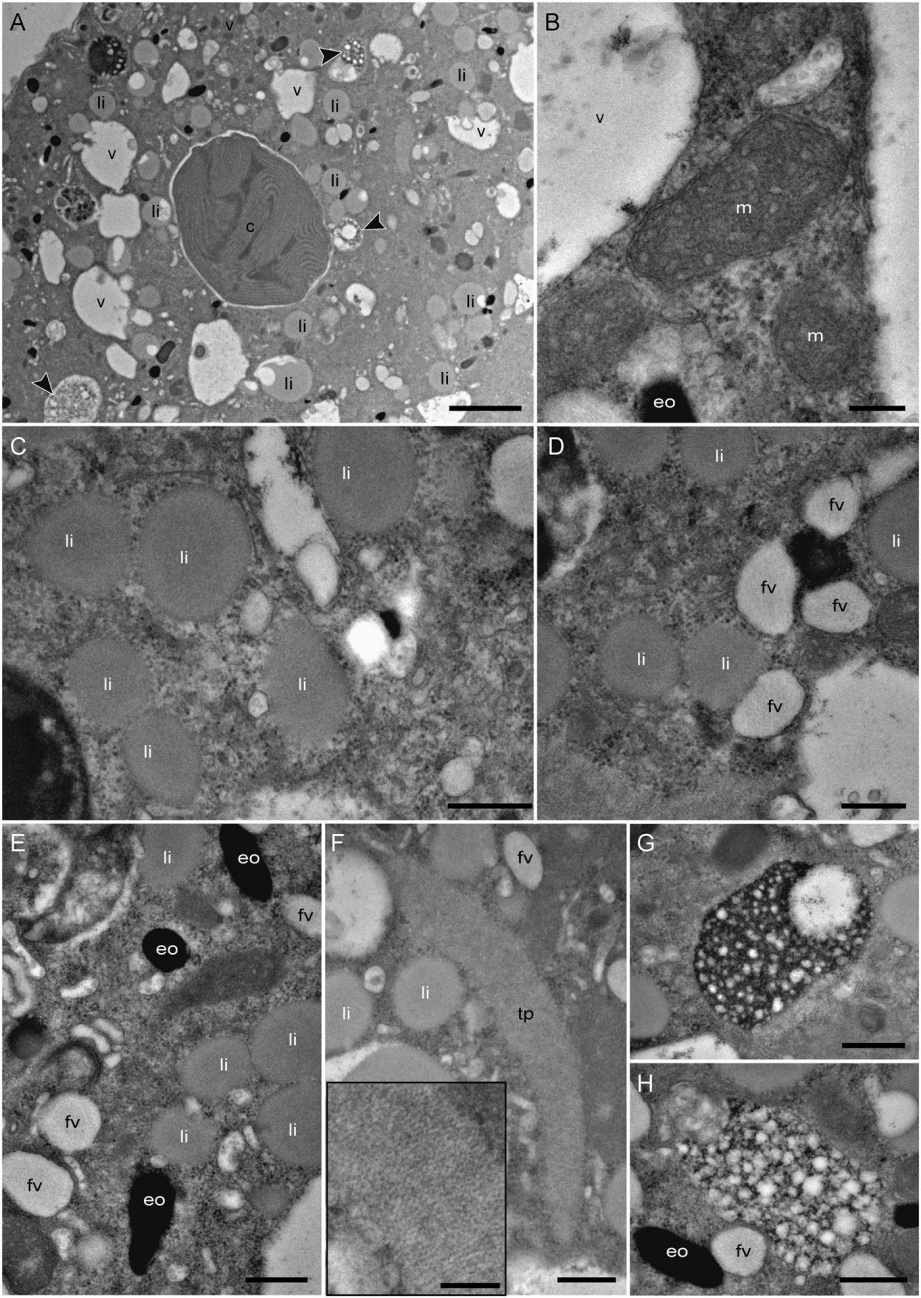
TEM micrographs of the cytoplasm and organelles of *Haynesina germanica*. (**A**) Aspect of the cytoplasm in a chamber of the penultimate whorl. (**B**) Intact mitochondria with well-defined cristae and intact double-membranes. (**C**) Small lipid droplets. (**D**) Fibrillar vesicles. (**E**) Electron-opaque bodies, (**F**) Tubulin paracrystals; Inset: higher magnification revealing regular pattern of the paracrystal ultrastructural organization. (**G**,**H**) multi-vacuolar structures. Arrowheads: multi-vacuolar structures; c: chloroplast; eo: electron-opaque bodies, fv: fibrillar vesicles, li: lipid droplets; m: mitochondria, tp: tubulin paracrystals, v- vacuole. Scale bars: A: 2 µm; B, inset F: 200 nm; C–H: 500 nm.

The cytoplasm of all specimens had well-preserved ultrastructure (Fig. 2A), as well as intact mitochondria with visible double-membranes and cristae (Fig. 2B). Numerous small lipid droplets (diameter of ca. 500 nm), recognized by their waxy appearance, were observed in the cytoplasm (Fig. 2C), along with some larger lipid droplets ranging from 1 to 3 µm in diameter. Numerous small oval fibrillar vesicles (ca. 500 nm in length), with the fibrils arranged in parallel, and spherical to oval-shaped electron-opaque bodies (200–500 nm) were observed in the cytoplasm (Fig. 2D,E), along with occasional tubulin paracrystals identifiable due to the regular pattern of their ultrastructure revealed by high-magnification TEM imaging (Fig. 2F). In all specimens, we observed many small structures (2 to 3 µm in length) variable in shape but mainly ovoid (Fig. 2A) with the presence of numerous vacuoles within their matrix (Fig. 2G,H). Henceforth, we refer to these as “multi-vacuolar structures”.

**Figure 3:**
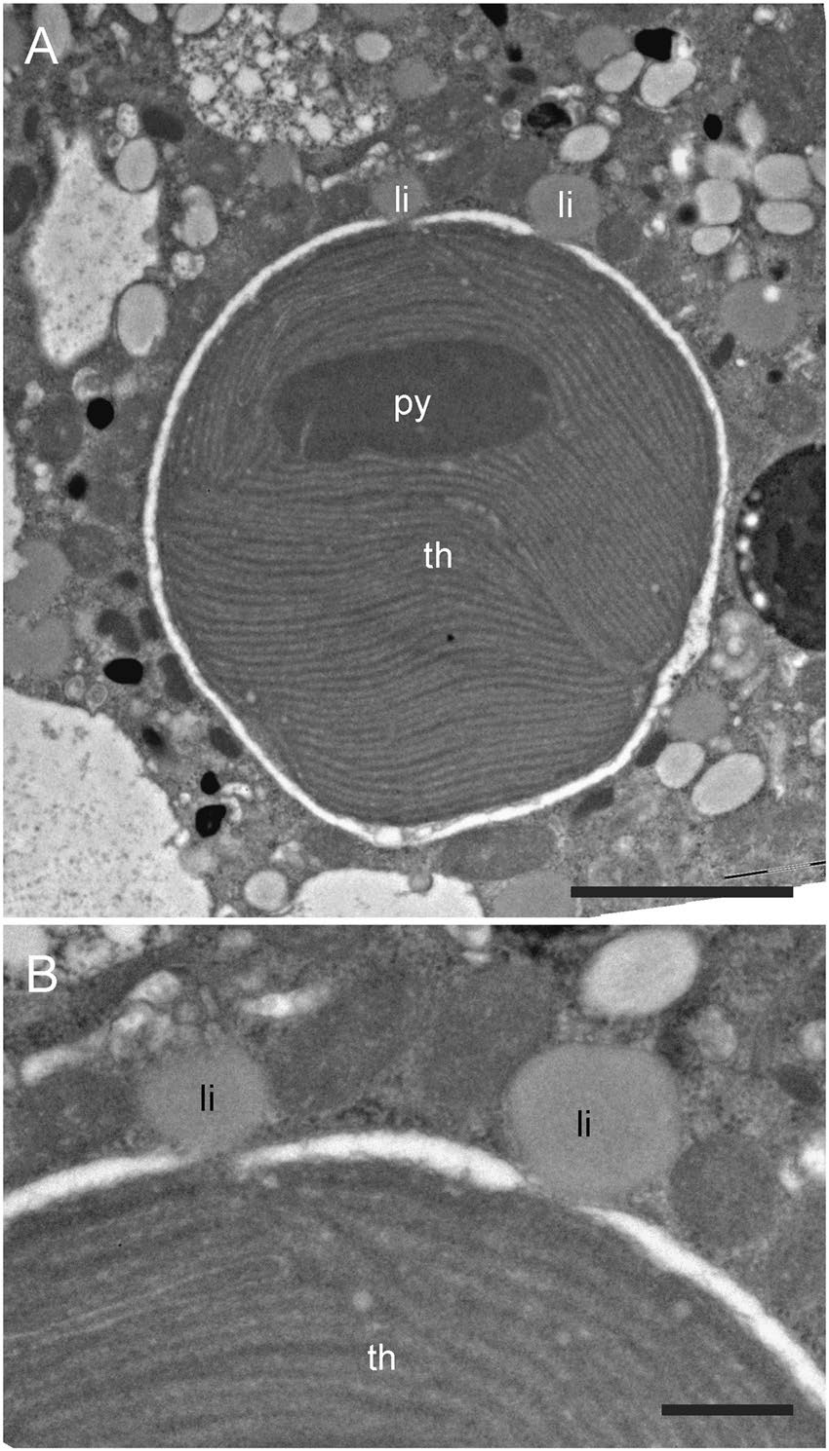
TEM micrographs of one chloroplast in *Haynesina germanica* cytoplasm. (**A**) Intact pyrenoid and thylakoids. (**B)** Higher magnification image showing two small lipid droplets in contact with the chloroplast membranes. The chloroplast membranes adjacent to the lipid vesicle are disrupted. li: lipid droplets, py: pyrenoid, th: thylakoid. Scale bars: A: 2 µm; B: 500 nm.

**Figure 4:**
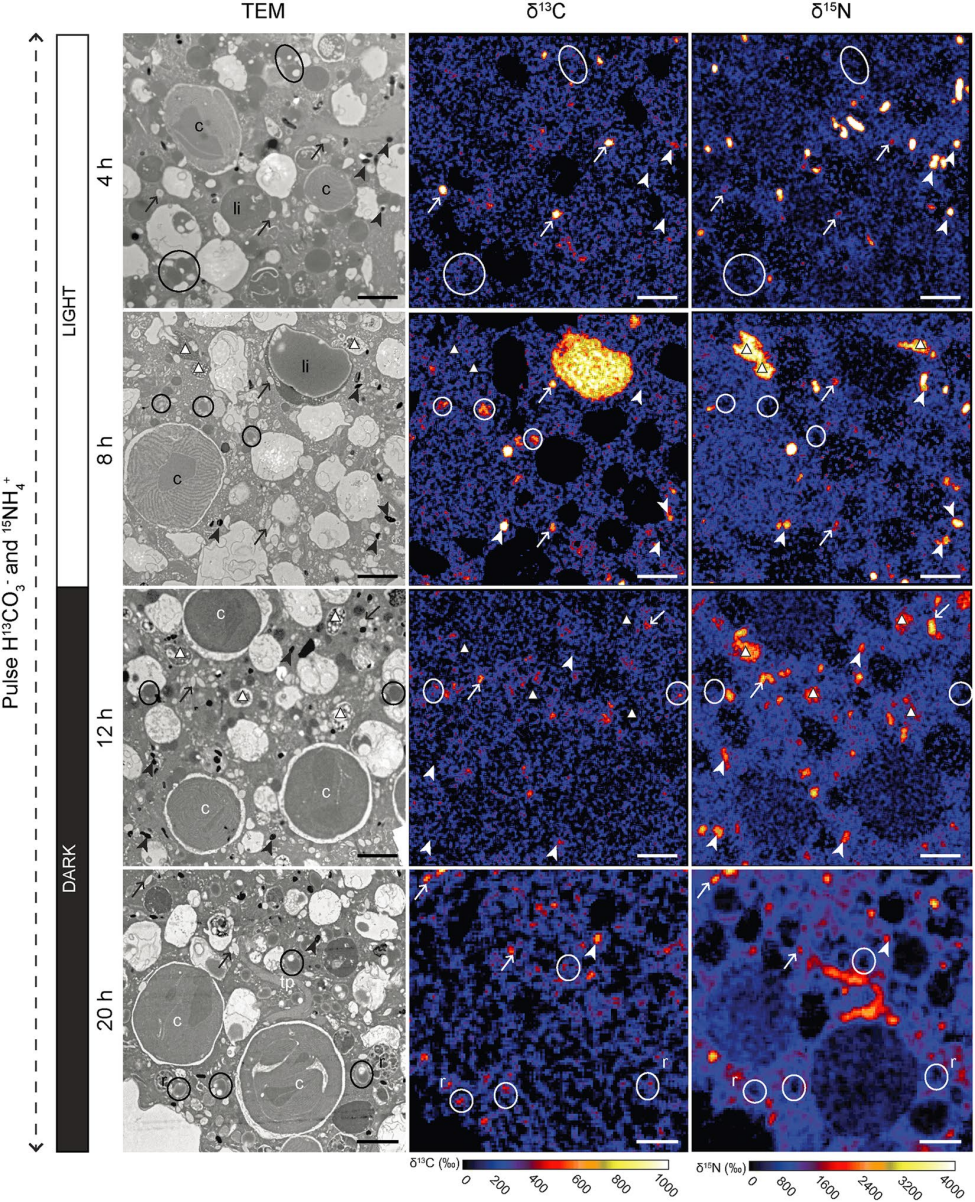
Time-evolution of ^13^C and ^15^N uptake and localization within the cytoplasm of *H*. *germanica* during Experiment 1 (light/dark incubation with H^13^CO_3_^−^ and ^15^NH_4_^+^). Left column: TEM micrographs. Middle and right columns: corresponding NanoSIMS δ^13^C and δ^15^N images, respectively, expressed in ‰. Arrows: fibrillar vesicles; arrowheads: electron opaque bodies; circles and li: lipid droplets, white triangles: multi-vacuolar structures; c: chloroplast; tp: tubulin paracrystals. Scale bars: 2 µm.

In all observed specimens, TEM images of the endoplasm revealed well-preserved kleptoplasts with visible pyrenoids and thylakoids (Figs 2A, 3, 4). These kleptoplasts ranged in size from 2 to 10 µm in diameter. Generally, their outlines were circular to oval. They were distributed in the endoplasm with no clear pattern and often surrounded by an electron-lucent space between the kleptoplast membranes and the endoplasm. Some of the small lipid droplets were observed adjacent to the kleptoplast periphery. In some cases, lipid droplets were even closely associated with kleptoplast membranes (Fig. 3B).

### Uptake of H^13^CO_3_^−^ within foraminiferal cells

**Figure 5:**
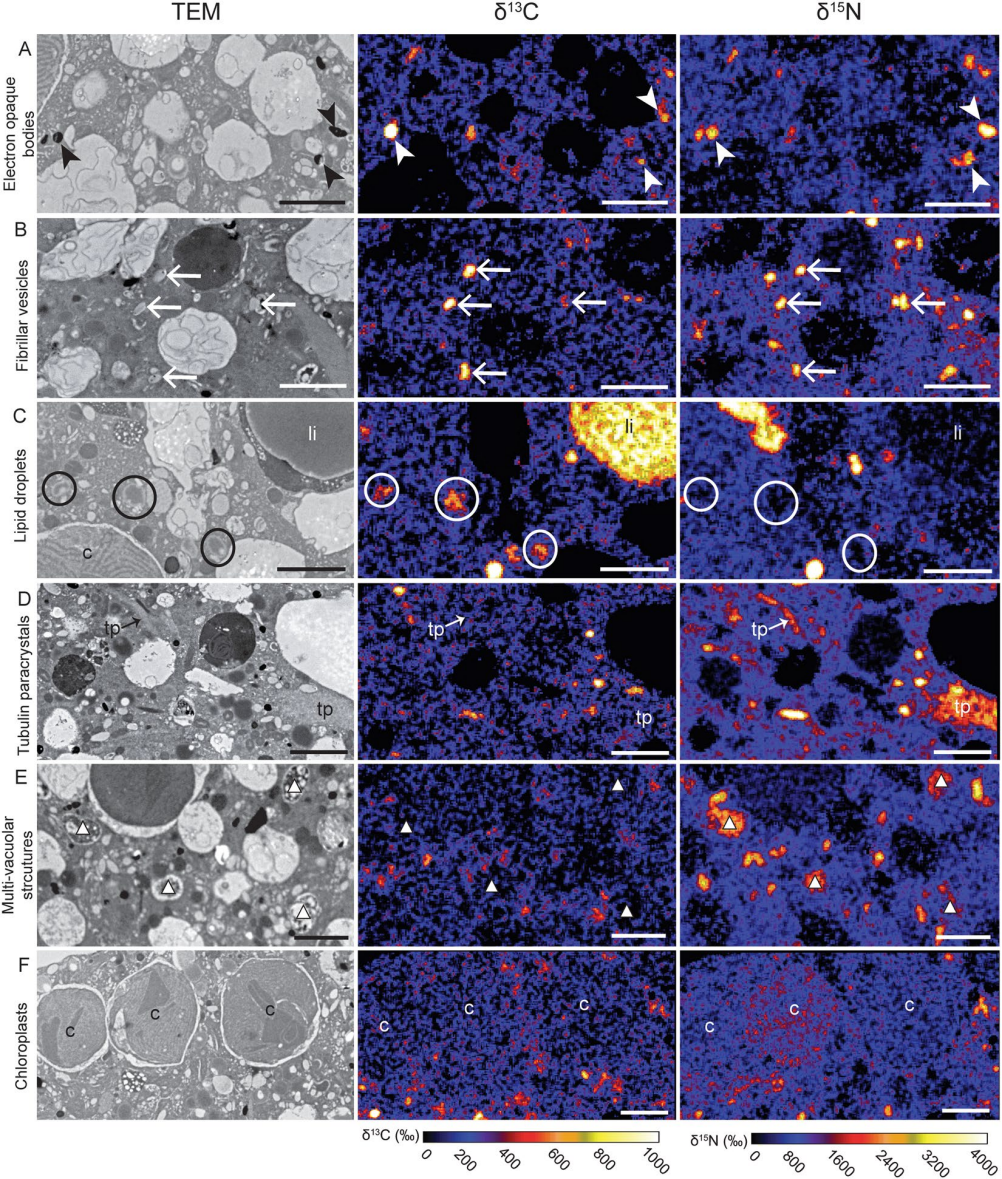
Foraminiferal organelles enriched in ^13^C and/or ^15^N in Experiment 1 at different time points. Left column: TEM micrographs. Middle and right columns: corresponding NanoSIMS δ^13^C and δ^15^N images, respectively, expressed in ‰. (**A**) electron-opaque bodies (after 8 h of incubation), (**B**) fibrillar vesicles (after 12 h of incubation), (**C**) lipid droplets (after 8 h of incubation), (**D**) tubulin paracrystals (after 20 h of incubation), (**E**) multi-vacuolar structures (after 12 h of incubation), (**F**) chloroplasts (after 8 h of incubation). Arrowheads: electron-opaque bodies; arrows: fibrillar vesicles; circles and li: lipid droplets; c: chloroplasts; white triangles: multi-vacuolar structures; c: chloroplast; tp: tubulin paracrystals. Scale bars: 2 µm.

**Figure 6:**
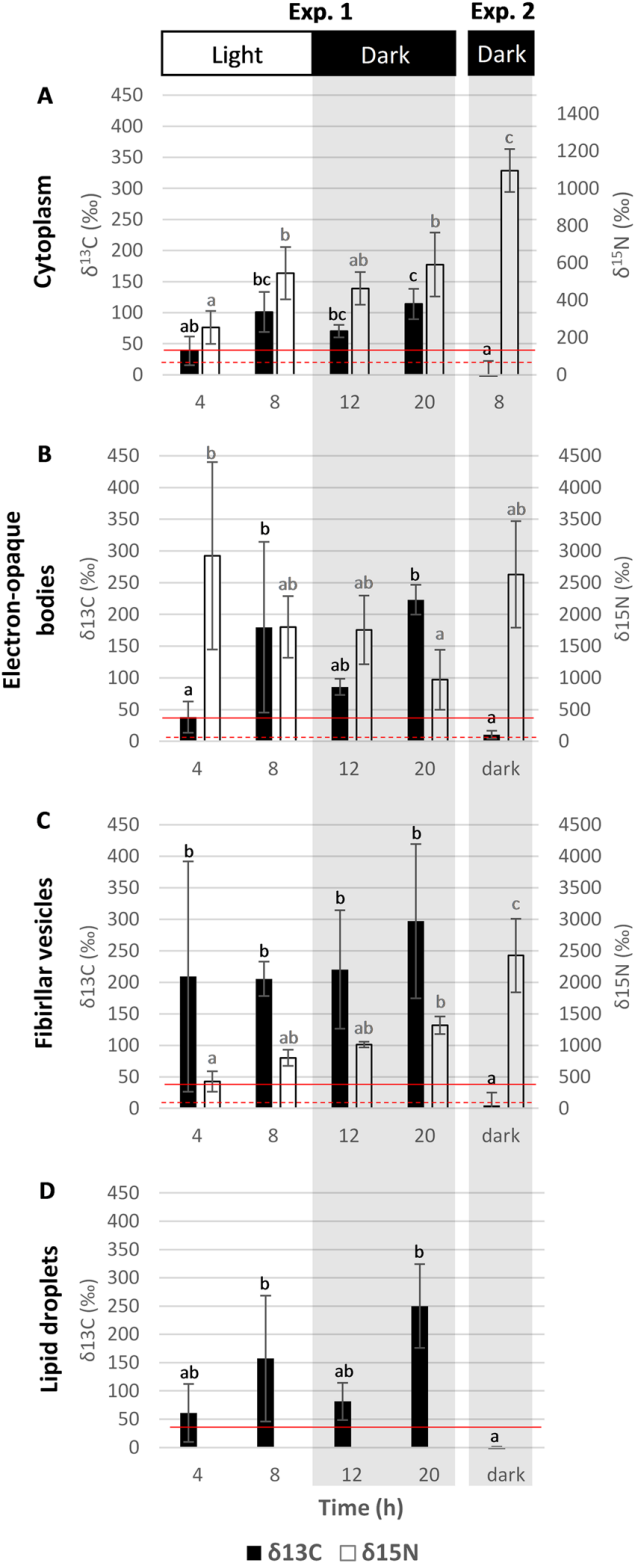
Average ^13^C (black bars) and ^15^N (white bars) enrichment of (**A**) the cytoplasm, (**B**) electron-opaque bodies, (**C**) fibrillar vesicles and (**D**) lipid droplets of *H*. *germanica* (n = 3) in Experiments 1 and 2. Error bars represent one standard deviation. Red lines indicate natural variations in ^13^C (solid lines) and ^15^N (dotted lines) enrichments as measured by NanoSIMS in similar areas of unlabelled control *H*. *germanica* specimens (n = 3; δ^13^C = 0 ± 40‰, and δ^15^N = 0 ± 60‰, 3*σ*).

**Figure 7:**
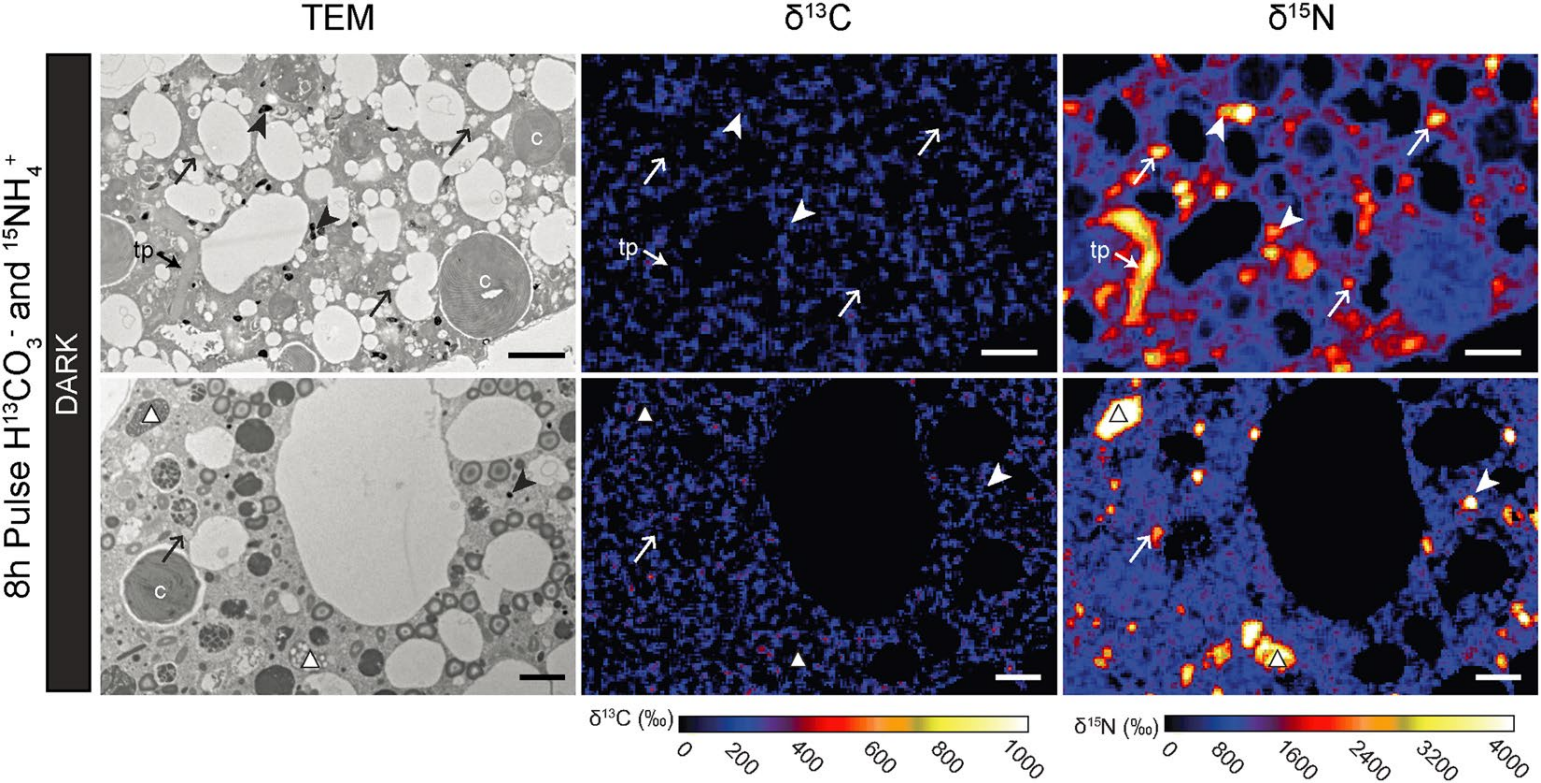
^13^C and ^15^N uptake and localization within the cytoplasm of *H*. *germanica* during Experiment 2 (continuous dark incubation with H^13^CO_3_^−^ and ^15^NH_4_^+^). Left column: TEM micrographs. Middle and right columns: corresponding NanoSIMS δ^13^C and δ^15^N images, respectively, expressed in ‰. Arrows: fibrillar vesicles; arrowheads: electron opaque dense bodies; circles: lipid droplets, white triangles: multi-vacuolar structures; c: chloroplast, tp: tubulin paracrystals. Scale bars: 2 µm.

In Experiment 1, starting at t = 8 h, ^13^C-enrichments were detected in all specimens. The signal was concentrated in fibrillar vesicles, electron opaque bodies, and lipids (Figs 4, 5A,B,C). In contrast, only one specimen from the first time point (*i*.*e*. at 4 h) exhibited ^13^C-enriched structures, concentrated in fibrillar vesicles and electron opaque bodies (Fig. 4). Although some lipid droplets were present, they were only slightly enriched at 4 h (Figs 4 and 6). All specimens collected between 8 and 20 h of incubation exhibited ^13^C-enrichments in the endoplasm. The ^13^C-enrichment (expressed in δ^13^C) of electron-opaque bodies significantly increased during the light phase from ca. 40‰ at 4 h to 180‰ at 8 h (*p* < 0.05) and remained stable through the dark phase of the experiment, i.e. between 8 and 20 h of incubation (Fig. 6B). The ^13^C-enrichments of the fibrillar vesicles and the lipid droplets were relatively stable during the incubation at ca. 200 to 300‰ and 60 to 150‰, respectively (Fig. 6C,D). The cytoplasm itself was slightly more enriched after 8 h of incubation than after 4 h, with averages of ca. 100‰ and 40‰, respectively (Fig. 6A). However, the cytoplasmic enrichment did not change statistically between 8 h and 20 h (*p* > 0.05; Fig. 6A). No ^13^C-enrichments were found in foraminifera incubated with H^13^CO_3_^−^ in darkness (Experiment 2; Figs 6 and 7).

### Uptake of ^15^NH_4_^+^ in the foraminiferal cell

All specimens of Experiment 1 exhibited detectable ^15^N-enrichments. In the cytoplasm of *H*. *germanica*, ^15^N-enrichments significantly increased between 4 and 8 h (during the light phase), from ca. 250 to 550‰ (*p* < 0.05), and stabilized between 8 and 20 h (*p* > 0.05), i.e., during the dark phase (Fig. 6A). Similar to the observed ^13^C-enrichments, the ^15^N-signal was concentrated in electron-opaque bodies and fibrillar vesicles (Figs 4, 5A,B and 6). The ^15^N-enrichment of electron-opaque bodies was relatively stable from 4 to 12 h (between 1800–2900‰) and then decreased to less than 1000‰ at 20 h (*p < *0.05 between 4 and 20 h; Fig. 6B). At the same time, the ^15^N-enrichment of the fibrillar vesicles increased throughout the incubation passing from ca. 425‰ at 4 h to 1320‰ after 20 h (*p* < 0.05). Some of these organelles were occasionally enriched in ^15^N but not in ^13^C. The tubulin paracrystals and the multi-vacuolar structures were also strongly enriched in ^15^N after 8 h (Fig. 5D,E). Kleptoplasts rarely exhibited ^15^N-enrichments, and if such enrichments were observed, they were always moderate to low (Fig. 5F).

In Experiment 2, after 8 h in darkness, the foraminifera had incorporated a much higher concentration of ^15^NH_4_^+^ (Fig. 6) compared with Experiment 1 at any given time (*p* < 0.05); the cytoplasmic average ^15^N-enrichment reached a value of ca. 1100‰ after 8 h of incubation in darkness. The fibrillar vesicles were also significantly more ^15^N-enriched during the second experiment reaching values of ca. 2400‰ (*vs*. maximum of ca. 1300‰ during Experiment 1). In contrast, the electron-opaque bodies exhibited ^15^N-enrichment comparable to those recorded during the first experiment, i.e. around ca. 2600‰. As in Experiment 1, the ^15^N isotopic signal was observed most concentrated in electron-opaque bodies, fibrillar vesicles, tubulin paracrystals, in the multi-vacuolar structures, as well as in a few kleptoplasts (Figs 6 and 7).

## Discussion

All specimens exhibited mitochondria with intact cristae and double-membranes indicating that they were alive at the time of fixation^42,44^. The kleptoplasts observed in our study correspond to the morphological description of^10^ for *H*. *germanica* collected from the Bourgneuf Bay (as in this study) and from the Wadden Sea (Mokbaai, NL). Specimens were well preserved with undamaged thylakoids and pyrenoids. The electron-lucent space that sometimes surrounded the kleptoplasts was also previously described by^10^ and ascribed to a possible fixation artefact. Indeed, TEM observations of the same species fixed using high pressure freezing and freeze substitution instead of “classic” chemical fixation revealed kleptoplasts without electron-lucent space, with their membranes directly in contact with the surrounding foraminiferal cytoplasm^16,17^.

The paired TEM-NanoSIMS observations allowed the visualization of inorganic C uptake (H^13^CO_3_^−^) within foraminiferal cells incubated under a light/dark cycle (Figs 4 and 6). The absence of ^13^C assimilation in continuous darkness (Experiment 2, Figs 6 and 7) and the observed production of O_2_ under light, as observed for *H*. *germanica* in other studies^11,23^, strongly suggests that *H*. *germanica* kleptoplasts have a functional Calvin-Benson cycle, resulting in the production and transfer of ^13^C-photosynthates to the *H*. *germanica* cell. Foraminifera can acquire C by different trophic mechanisms^45^, but they are not known to actively uptake inorganic C in the absence of either bacterial or algal symbionts or in the presence of kleptoplasts. We found no indications of the presence of prokaryotic symbiotic photosynthetic organisms and, therefore, suggest that the observed incorporation of ^13^C-bicarbonate is the result of photosynthesis occurring in the kleptoplasts. However, the absence of ^13^C-enrichment inside the kleptoplasts (Figs 4 and 5) was unexpected. This absence of ^13^C-enrichment can be attributed to the fact that the ^13^C-photosynthates are quickly transported away from the kleptoplasts and thus the ^13^C-enrichment stay below the detection limit of the NanoSIMS. This hypothesis is supported by previous NanoSIMS studies of autotrophic ^13^C-exchanges in the symbiotic association between dinoflagellates and corals, where ^13^C-enrichments in dinoflagellate chloroplasts were systematically much lower than in other sub-cellular organelles^34^. Additionally, studies on *H*. *germanica* have shown that the cellular machinery necessary for chloroplast maintenance is unlikely to be functional^23^, which could explain why, in our Experiment 1, the kleptoplasts did not accumulate ^13^C within their structures. In summary, our observations show that kleptoplasts in *H*. *germanica* are able to assimilate inorganic C and form ^13^C-photosynthates that are transferred to the host cell, but the kleptoplasts do not themselves become enriched in ^13^C (above the detection limit of the NanoSIMS).

The numerous multi-vacuolar structures observed in *H*. *germanica* (Fig. 2A,G,H) are somewhat similar to the bacteria observed in another benthic species, *Globocassidulina* cf. *G*. *biora*^46^. The presence of numerous such vacuoles within prokaryotic cells has been described and linked to different biological functions, such as buoyancy (gas vacuoles) in planktonic bacteria or nitrate vacuoles in filamentous sulfur bacteria^47,48^. Thus these structures could potentially be interpreted as endobionts. Is it noteworthy that they were not labeled in ^13^C (Fig. 5E). Other NanoSIMS studies looking at ^13^C-bicarbonate assimilation in cyanobacteria, anaerobic photosynthetic bacteria or chemotrophic bacteria have shown strong bacterial ^13^C-enrichments^49–52^. Therefore, even if the multi-vacuolar structures observed in our study were bacteria, the absence of ^13^C incorporation into their structure suggests that they are not photosynthesizing or assimilating inorganic carbon and that thus they do not play any role in the inorganic ^13^C assimilation in *Haynesina germanica*.

Carbon was assimilated during the light phase, transferred to the foraminiferal cell, and accumulated in specific organelles: electron-opaque bodies, fibrillar vesicles, and lipid droplets. The ^13^C-assimilation dynamics in electron-opaque bodies and fibrillar vesicles should be interpreted with caution, as these organelles are poorly understood^44^. Indeed, we do not know their function(s) in the cell, how fast they are produced, their turn over and what triggers their production. In addition, we do not know if the ^13^C-enriched hotspots observed within the foraminiferal endoplasm correspond only to newly formed electron-opaque bodies and fibrillar vesicles, or if ^13^C-enriched material was added to pre-existing organelles; which is likely considering the high variability observed for enrichment values (Fig. 6). However, we note that none of these organelles exhibited an increase in their ^13^C-enrichment during the dark phase of Experiment 1, and none of the analyzed organelles showed ^13^C-enrichment during Experiment 2. This strongly indicates that there are no cytoplasmic foraminiferal pathways for inorganic carbon assimilation; i.e. carbon is assimilated only via the photosynthetic kleptoplasts.

Lipid droplets are considered to be the main C storage form in foraminifera^44^. A similar accumulation process/sequence has been observed in the symbiotic planktonic foraminifer *Orbulina universa*, where photosynthesis led to an assimilation of inorganic C (H^13^CO_3_^−^) stored in the form of lipid droplets^43^. In kleptoplastic sea slugs (e.g., *Elysia chlorotica*), lipid droplets observed in the animal tissue where argued to result from the exudation of lipids from the plastids because their fatty acids had a large proportion of algal-derived eicosapentaenoic acid (20:5)^53^. However, these authors could not determine whether the plastids transferred fatty acids directly via triacylglycerols (TAGs), or as free fatty acids that may be further transformed by the host into lipid droplets. The *de novo* production of triacylglycerol by chloroplasts in marine algae has been demonstrated^54,55^. Furthermore, *de novo* fatty acid synthesis is known to occur in plant cell chloroplasts^56^, followed by a transfer in the form of free fatty acids to the cytosol^57^. Additional transfer of soluble molecules such as maltose or glucose across the chloroplast membranes through transporters also occurs in plant cells^58^. The close spatial association between kleptoplast membranes and small lipid droplets observed here (Fig. 3) may indicate a potential transfer of C via exudation of small lipid droplets from kleptoplast to the *H*. *germanica* cell, although the detailed mechanisms by which the fatty acids cross kleptoplast membranes remain unknown. Unfortunately, the distribution of soluble molecules cannot be investigated with NanoSIMS because the sample preparation protocol causes near complete loss of such components^41^.

Teugels *et al*.^59^ reported that ammonium assimilation by the kleptoplastic sacoglossan *Elysia viridis* was significantly higher during light exposure than in darkness. This is consistent with the glutamine oxoglutarate aminotransferase (GOGAT) enzyme pathway that requires electron donors (e.g., reduced ferredoxin) formed during photosynthetic electron transport^60^. Furthermore, the glutamate synthase (GS) metabolic reaction is ATP-dependent, and gene expression of some key enzymes (GS and GOGAT) is light regulated^60^. In corals, symbiotic dinoflagellate GS/GOGAT enzymes are thought to be the main ammonium assimilation pathway^35,38,61,62^. Furthermore, cnidarian cells are also known to produce cytosol glutamate dehydrogenase (GDH)^62–64^, which has a dual function: 1) it converts glutamate to α-ketoglutarate and ammonium that is subsequently assimilated into chloroplasts via the GOGAT pathway^59^; 2) it catalyzes the opposite reaction, i.e. the amination of the α-ketoglutarate to produce the amino acid glutamate^65^.

In our study, the observation of ^15^N-labeled kleptoplasts in *H*. *germanica* incubated both in light and in darkness seems consistent with a GS/GOGAT kleptoplastic pathway for ammonium assimilation (Fig. 5H). However, the uptake of ^15^N-ammonium was higher after 8 h of incubation in total darkness (Experiment 2) than in Experiment 1 (light-dark cycle) (Fig. 6). This higher uptake in darkness compared to light is inconsistent with the light regulation of the GS/GOGAT enzymatic machinery^60^. Ammonium incorporation might, thus, also occur by another N-assimilation pathway in foraminifera, for example, through the glutamine dehydrogenase (GDH) pathway.

The same organelles (fibrillar vesicles, electron-opaque bodies and multi-vacuolar structures) were found to be ^15^N-enriched in both Experiments 1 and 2. As previously noted for ^13^C-assimilation, the ^15^N-assimilation dynamics in electron-opaque bodies and fibrillar vesicles is difficult to interpret, due to the lack of knowledge about the function of these organelles^44^. However, the electron-opaque bodies and the fibrillar vesicles seem to have different patterns for ^15^N assimilation. While the electron-opaque bodies incorporated large amounts of ^15^N even after only 4 hours and then gradually lost this ^15^N-enrichment over time, the fibrillar vesicles assimilated ^15^N throughout the 20 h incubation, independent of light condition. It is noteworthy that the ^15^N-assimilation pattern clearly differs from the ^13^C-assimilation dynamics in both organelle types.

Finally, ammonium is known to be a suitable N source for many marine prokaryotes^66–68^. Thus, if the multi-vacuolar structures, abundant in all *H*. *germanica* specimens (Fig. 2A,G,H) are endosymbionts (see above) they would be expected to incorporate ^15^NH_4_^+^, as is indeed observed (Fig. 5G). They could, thus, constitute another putative nitrogen assimilation pathway for the benthic foraminifer *H*. *germanica*. However, we cannot conclude further in this study about the symbiotic nature of these putative prokaryotes in *H*. *germanica*, nor about their role in foraminiferal N metabolism.

A comparative study of organic C (algae) uptake through feeding between the two dominant foraminiferal species inhabiting mudflats, the akleptoplastic *Ammonia* sp. and kleptoplastic *H*. *germanica*, showed a higher uptake rate by the former^69^. Our results highlight that *H*. *germanica* can fix inorganic carbon. Therefore, unlike *Ammonia* sp., *H*. *germanica* does not rely solely on heterotrophy to meet its C requirements. The mixotrophic feeding strategy of *H*. *germanica* might give a competitive advantage and allow it to become the dominant foraminifera in mudflat environments^70–72^. In addition, whether *H*. *germanica* assimilates nitrogen through the kleptoplasts, potential endosymbionts, and/or by another pathway specific to foraminifera, our observations demonstrate that it is also capable of using inorganic N as a nutrient source. Further investigation is required to quantify this uptake and elucidate the role of this benthic foraminifera in the N cycle, especially since *H*. *germanica* thrives in coastal ecosystems that are subject to increasing eutrophication^73,74^.

## Conclusion

Our study demonstrates inorganic C assimilation in *H*. *germanica*, most likely via the kleptoplasts. The absence of ^13^C assimilation in darkness combined with previous studies documenting O_2_ production in light strongly suggest that photosynthesis is the process dominating inorganic C-assimilation in this species. Subsequently, photosynthates are transferred to the foraminiferal cell and utilized for its metabolism. Thus, these observations clearly show the role played by the kleptoplasts in *H*. *germanica* carbon metabolism, providing the foraminiferal cell with an additional autotrophic source of C. The observation of small lipid droplets attached to the kleptoplast membranes may suggest a transfer of C from the kleptoplasts to the foraminiferal cell in the form of lipids. However, the detailed mechanism(s) involved in this C transfer remains unknown. The kleptoplasts may also provide additional N sources to foraminiferal metabolic pathways via GS-GOGAT enzymes. However, ammonium assimilation was more efficient in darkness, requiring the existence of other N-assimilation pathways.

## Material and Methods

### Experiment 1: light/dark cycle incubation with H^13^CO_3_^−^ and ^15^NH_4_^+^

Living foraminifera were collected on April 9, 2015, at low tide on the intertidal mudflat of the Bourgneuf Bay (France, 47°00′59.4″N 2°01′29.8″W). The top centimeter of the sediment was sampled, sieved over a mesh of 150 μm with *in situ* seawater and the >150 μm fraction was immediately transported in the dark over ∼3 hours to the laboratory in Nantes.

In the laboratory, healthy living individuals of *H*. *germanica* were selected under a binocular microscope based on their cytoplasm color (i.e. yellow-brownish material spread through all the chambers of the specimen, except the youngest chamber). The selected specimens were placed into 5 Petri dishes (5 specimens per Petri dish) filled with artificial seawater (ASW, Red Sea Salt, salinity = 35, pH = 8.0). Four of the Petri dishes contained ASW enriched with 2 mM NaH^13^CO_3_ and 10 μM ^15^NH_4_Cl. The fifth Petri dish contained isotopically normal artificial seawater of the same chemical composition: specimens from this dish were fixed at T0 (beginning of the experiment) and served as controls for NanoSIMS analysis (see below). All other Petri dishes were placed in an incubator (Fytoscope FS130, temperature: 18 °C, light intensity: 90 μmol m^−2^ s^−1^). After 8 h of light exposure they were transferred to dark conditions for another 12 h. Except for the control specimens, the foraminifera remained in the spiked ASW throughout the experiment. At each time point, i.e., after 4, 8, 12, and 20 hours, one Petri dish was removed from the incubator (Fig. 1) and the 5 specimens contained in this Petri dish were immediately chemically fixed.

### Experiment 2: Incubation in continuous darkness with H^13^CO_3_^−^ and ^15^NH_4_^+^

*H*. *germanica* specimens were collected on May 16, 2015, at low tide on the intertidal mudflat of the Bourgneuf Bay (France) following the same procedure as described above. Five living specimens were selected and placed in a Petri dish with artificial seawater (Red Sea Salt, salinity = 35; pH = 8.0) enriched with 2 mM NaH^13^CO_3_ and 10 μM of ^15^NH_4_Cl. They were incubated in continuous darkness for 8 h (Fig. 1) and immediately chemically fixed at the end of this incubation. Control samples, which were incubated in normal seawater, where fixed at the beginning of the experiment (T0; Fig. 1).

### Preparation for TEM-NanoSIMS analysis

The specimens were chemically fixed following the protocol described^44^). Briefly, foraminifera were fixed immediately after removal from the incubator, with a mix of 4% glutaraldehyde and 2% paraformaldehyde diluted in 0.1 M cacodylate buffer, 0.4 M sucrose, and 0.1 M NaCl (pH = 7.4) at room temperature for 24 h. They were then stored at 4 °C until further processing. Further chemical processing and transmission electron microscope (TEM) imaging of the foraminifera were performed at the Electron Microscopy Facility of the University of Lausanne (Switzerland). After rinsing, specimens were decalcified in two successive baths (1 and 48 h, respectively) with a solution of 0.1 M EDTA diluted in 0.1 M cacodylate buffer, then post-fixed for 1 h in 2% osmium tetroxide diluted in distilled water. After distilled water rinsing followed by serial dehydration in ethanol, the specimens were embedded into acrylic resin (LR White). Specimens were cut into 70 nm ultra-thin sections with an ultramicrotome (Reichert ultracut S), placed on carbon-formvar coated copper grids, and post-stained for 10 min with 2% uranyl acetate before observing with the TEM (Philips 301 CM100, 80 kV). Only chambers from *n* − 3 to *n* − 8 (*n* being the youngest chamber bearing the exposed aperture) were examined. The integrity of the mitochondria and membranes of each specimen were checked as recommended by^42^ to ensure the vitality of each individual.

### Stable isotope mapping with NanoSIMS

NanoSIMS analyses were conducted on areas defined on the basis of prior TEM observations. Grids with TEM sections were mounted on 10-mm disks with double stick Cu-tape and coated with a ca. 10-nm thick gold layer before being imaged with the NanoSIMS 50 L ion microprobe to image and quantify the distribution of ^13^C and ^15^N enrichment.

Foraminiferal sections were imaged with the NanoSIMS ion microprobe with a 16 keV primary ion beam of Cs^+^ focused to a beam spot of ca. 100–150 nm. The secondary molecular ions ^12^C_2_^−^, ^13^C^12^C^−^, ^12^C^14^N^−^ and ^12^C^15^N^−^ were collected simultaneously in electron multiplier detectors at a mass-resolution of ca. 10000, enough to resolve potential interferences in the mass spectrum^34,35^. Isotopic image dimensions ranged from 15 × 15 µm to 30 × 30 µm with 256 × 256 pixel resolution. For each image, 6 layers were acquired, drift corrected and superimposed using the software L’IMAGE (developed by Dr. Larry Nittler, Carnegie Institution of Washington DC, USA). Quantified ^13^C/^12^C and ^15^N/^14^N ratios were obtained as follows:

$${\delta }^{13}C({\rm{\textperthousand }})=(({C}_{mes}/{C}_{nat})-1)\times {10}^{3}$$

$${\delta }^{15}N({\rm{\textperthousand }})=(({N}_{mes}/{N}_{nat})-1)\times {10}^{3}$$

where *C*_*mes*_ is the measured ^12^C^13^C^−^/^12^C_2_^−^ ratio of the sample and *C*_*nat*_ is the average ^12^C^13^C^−^/^12^C_2_^−^ ratio measured in unlabeled samples (control). Similarly, *N*_*mes*_ is the measured ^12^C^15^N^−^/^12^C^14^N^−^ ratio of the sample and *N*_*nat*_ is the average ^12^C^15^N^−^/^12^C^14^N^−^ ratio measured in unlabeled samples. The software Look@NanoSIMS^75^ was used to determine the isotopic enrichment of specific organelles that were identified morphologically from TEM images. Regions of interest (ROIs) to quantify the average isotopic enrichment of the organelles were defined from the TEM images previously aligned with the NanoSIMS images (based on the ^12^C^14^N image). For the average isotopic enrichment of the cytoplasm, three circles of ca. 2 μm in diameter were drawn per image, avoiding highly ^15^N-enriched organelles. For each specimen, between one and three NanoSIMS images were analyzed.

### Statistical analysis

For each time point, three specimens were analyzed. δ^13^C and δ^15^N values for main organelles and cytoplasm were obtained by calculating the average of ROIs within each specimen, and then calculating the average of the three specimens for each time point. The error bars provided are thus standard deviations representing inter-specimen variability. However, for the statistical analysis, a linear mixed-effects (LME) model was constructed using all the ROIs of the three specimens for each time point (taking into account pseudo-replication effects, i.e. regrouping ROIs from three different specimens into one category), followed by a Tukey multiple comparison test. The results of the Tukey multiple comparisons tests are given in the Supplementary Data S1. The statistical analyses were performed with Rstudio software using a significance level set at *α* = 0.05.

### Data Availability

The datasets generated and/or analyzed during the current study are available in the PANGAEA Repository (10.1594/PANGAEA.891407 data will be published upon acceptance of the manuscript).

## Electronic supplementary material

Supplementary material
