# Supplementary material for: Inorganic carbon and nitrogen assimilation in cellular compartments of a benthic kleptoplastic foraminifer
Source: Sci Rep. 2018 Jul 4;8:10140. doi: 10.1038/s41598-018-28455-1 (PMC6031614; doi:10.1038/s41598-018-28455-1)
Supplement: Supplementary file 1 — Supplementary material [file 41598_2018_28455_MOESM1_ESM.pdf]

Inorganic carbon and nitrogen assimilation in cellular compartments of a benthic kleptoplastic foraminifer

Charlotte LeKieffre, Thierry Jauffrais, Emmanuelle Geslin, Bruno Jesus, Joan M. Bernhard, Maria-Evangelia Giovani, Anders Meibom

Supplementary material 1: Results of the different post-hoc Tukey multiple comparisons tests following the linear mixed-effects model analyses.

Note: “4, 8, 12, 20 and dark” in the columns “Time points” correspond to the time points 4, 8, 12, and 20 h of the Experiment 1, and to the time point 8 h of the Experiment 2, respectively.

- δ^{13}C cytoplasm

```
       Simultaneous Tests for General Linear Hypotheses

Multiple Comparisons of Means: Tukey Contrasts

Fit: lme.formula(fixed = Y ~ time, data = mydata, random = ~1 | Plotb)

Linear Hypotheses:
Time points      Estimate Std. Error z value Pr(>|z|)
20 - 12 == 0       45.6       24.0    1.90   0.3166
4 - 12 == 0       -32.7       24.3   -1.35   0.6619
8 - 12 == 0        31.3       24.0    1.31   0.6876
dark - 12 == 0    -71.9       25.3   -2.85   0.0357 *
4 - 20 == 0       -78.3       24.0   -3.27   0.0095 **
8 - 20 == 0       -14.3       23.6   -0.60   0.9746
dark - 20 == 0   -117.5       24.9   -4.71   <0.001 ***
8 - 4 == 0         64.0       24.0    2.67   0.0585 .
dark - 4 == 0     -39.2       25.3   -1.55   0.5289
dark - 8 == 0    -103.2       24.9   -4.14   <0.001 ***
---
Signif. codes:  0 ‘***’ 0.001 ‘**’ 0.01 ‘*’ 0.05 ‘.’ 0.1 ‘ ’ 1
(Adjusted p values reported -- single-step method)
```

- δ^{15}N cytoplasm

```
       Simultaneous Tests for General Linear Hypotheses

Multiple Comparisons of Means: Tukey Contrasts

Fit: lme.formula(fixed = Z ~ time, data = mydata, random = ~1 | Plotb)

Linear Hypotheses:
Time points      Estimate Std. Error z value Pr(>|z|)
20 - 12 == 0      137.4      102.8    1.34   0.6677
4 - 12 == 0      -201.5      104.2   -1.93   0.2991
8 - 12 == 0        91.6      102.8    0.89   0.9002
dark - 12 == 0    638.7      108.5    5.88   <0.001 ***
4 - 20 == 0      -339.0      102.8   -3.30   0.0085 **
8 - 20 == 0       -45.8      101.3   -0.45   0.9914
dark - 20 == 0    501.2      107.1    4.68   <0.001 ***
8 - 4 == 0        293.1      102.8    2.85   0.0350 *
dark - 4 == 0     840.2      108.5    7.74   <0.001 ***
dark - 8 == 0     547.1      107.1    5.11   <0.001 ***
---
Signif. codes:  0 ‘***’ 0.001 ‘**’ 0.01 ‘*’ 0.05 ‘.’ 0.1 ‘ ’ 1
(Adjusted p values reported -- single-step method)
```

- <u>$\delta^{13}C$ electron-opaque bodies</u>

```
       Simultaneous Tests for General Linear Hypotheses

Multiple Comparisons of Means: Tukey Contrasts

Fit: lme.formula(fixed = C ~ time, data = mydata, random = ~1 | Plotb)

Linear Hypotheses:
Time points      Estimate Std. Error z value Pr(>|z|)
20 - 12 == 0      132.9       51.8    2.56   0.0768 .
4 - 12 == 0       -49.5       50.0   -0.99   0.8598
8 - 12 == 0        92.1       49.6    1.86   0.3406
dark - 12 == 0    -75.3       49.8   -1.51   0.5547
4 - 20 == 0      -182.4       53.7   -3.40   0.0061 **
8 - 20 == 0       -40.8       53.3   -0.77   0.9404
dark - 20 == 0   -208.2       53.5   -3.89   <0.001 ***
8 - 4 == 0        141.6       51.5    2.75   0.0475 *
dark - 4 == 0     -25.8       51.8   -0.50   0.9875
dark - 8 == 0    -167.4       51.4   -3.26   0.0098 **
---
Signif. codes:  0 '***' 0.001 '**' 0.01 '*' 0.05 '.' 0.1 ' ' 1
(Adjusted p values reported -- single-step method)
```

- <u>$\delta^{15}N$ electron-opaque bodies</u>

```
       Simultaneous Tests for General Linear Hypotheses

Multiple Comparisons of Means: Tukey Contrasts

Fit: lme.formula(fixed = N ~ time, data = mydata, random = ~1 | Plotb)

Linear Hypotheses:
Time points       Estimate Std. Error z value Pr(>|z|)
20 - 12 == 0       -792        689   -1.15    0.780
4 - 12 == 0        1133        685    1.65    0.462
8 - 12 == 0          48        683    0.07    1.000
dark - 12 == 0      882        684    1.29    0.698
4 - 20 == 0        1925        694    2.78    0.044 *
8 - 20 == 0         840        692    1.21    0.743
dark - 20 == 0     1674        692    2.42    0.111
8 - 4 == 0        -1085        688   -1.58    0.512
dark - 4 == 0      -251        688   -0.36    0.996
dark - 8 == 0       834        687    1.21    0.743
---
Signif. codes:  0 '***' 0.001 '**' 0.01 '*' 0.05 '.' 0.1 ' ' 1
(Adjusted p values reported -- single-step method)
```

- $\delta^{13}C$ fibrillar vesicles

```
Simultaneous Tests for General Linear Hypotheses

Multiple Comparisons of Means: Tukey Contrasts


Fit: lme.formula(fixed = C ~ time, data = mydata, random = ~1 | Plotb)

Linear Hypotheses:
Time points       Estimate Std. Error z value Pr(>|z|)
20 - 12 == 0       64.82      63.91    1.01   0.8460
4 - 12 == 0        71.09      86.30    0.82   0.9217
8 - 12 == 0        -9.74      62.25   -0.16   0.9999
dark - 12 == 0  -208.71      63.86   -3.27   0.0094 **
4 - 20 == 0         6.27      88.19    0.07   1.0000
8 - 20 == 0       -74.57      64.84   -1.15   0.7759
dark - 20 == 0  -273.53      66.39   -4.12   <0.001 ***
8 - 4 == 0        -80.83      86.99   -0.93   0.8833
dark - 4 == 0   -279.80      88.16   -3.17   0.0125 *
dark - 8 == 0   -198.97      64.80   -3.07   0.0177 *
---
Signif. codes:  0 '***' 0.001 '**' 0.01 '*' 0.05 '.' 0.1 ' ' 1
(Adjusted p values reported -- single-step method)
```

- $\delta^{15}N$ fibrillar vesicles

```
Simultaneous Tests for General Linear Hypotheses

Multiple Comparisons of Means: Tukey Contrasts


Fit: lme.formula(fixed = N ~ time, data = mydata, random = ~1 | Plotb)

Linear Hypotheses:
Time points       Estimate Std. Error z value Pr(>|z|)
20 - 12 == 0         324        197    1.64    0.460
4 - 12 == 0         -519        293   -1.77    0.383
8 - 12 == 0         -218        190   -1.15    0.774
dark - 12 == 0      1469        197    7.45   <0.001 ***
4 - 20 == 0         -843        301   -2.80    0.039 *
8 - 20 == 0         -542        202   -2.69    0.053 .
dark - 20 == 0      1145        208    5.49   <0.001 ***
8 - 4 == 0           301        296    1.01    0.844
dark - 4 == 0       1988        301    6.60   <0.001 ***
dark - 8 == 0       1688        202    8.36   <0.001 ***
---
Signif. codes:  0 '***' 0.001 '**' 0.01 '*' 0.05 '.' 0.1 ' ' 1
(Adjusted p values reported -- single-step method)
```

- $\delta^{13}C$ lipid droplets

```
Simultaneous Tests for General Linear Hypotheses

Multiple Comparisons of Means: Tukey Contrasts


Fit: lme.formula(fixed = C ~ time, data = mydata, random = ~1 | Plotb)

Linear Hypotheses:
Time points       Estimate Std. Error z value Pr(>|z|)
20 - 12 == 0      148.4       64.6    2.30   0.1452
4 - 12 == 0       -20.4       57.7   -0.35   0.9966
8 - 12 == 0        76.1       57.5    1.32   0.6756
dark - 12 == 0    -87.7       65.3   -1.34   0.6632
4 - 20 == 0      -168.8       64.9   -2.60   0.0699 .
8 - 20 == 0       -72.3       64.8   -1.11   0.7979
dark - 20 == 0   -236.1       71.9   -3.29   0.0089 **
8 - 4 == 0         96.5       57.9    1.67   0.4522
dark - 4 == 0     -67.3       65.6   -1.03   0.8429
dark - 8 == 0    -163.8       65.5   -2.50   0.0899 .
---
Signif. codes:  0 '***' 0.001 '**' 0.01 '*' 0.05 '.' 0.1 ' ' 1
(Adjusted p values reported -- single-step method)
```
